# Increased risk of critical CBF levels in SAH patients with actual CPP below calculated optimal CPP

**DOI:** 10.1007/s00701-017-3139-7

**Published:** 2017-03-30

**Authors:** Ulf Johnson, Henrik Engquist, Anders Lewén, Tim Howells, Pelle Nilsson, Elisabeth Ronne-Engström, Elham Rostami, Per Enblad

**Affiliations:** 1 Department of Neuroscience/Neurosurgery, Uppsala University, S-75185 Uppsala, Sweden; 2 Department of Surgical sciences/Radiology, Uppsala University, Uppsala, Sweden; 3 Department of Surgical sciences/Anaesthesia and Intensive care, Uppsala University, Uppsala, Sweden

**Keywords:** Subarachnoid hemorrhage, SAH, Cerebral blood flow, CBF, Autoregulation, Optimal, Cerebral perfusion pressure, CPPopt, Neurointensive care

## Abstract

**Background:**

Cerebral pressure autoregulation can be quantified with the pressure reactivity index (PRx), based on the correlation between blood pressure and intracranial pressure. Using PRx optimal cerebral perfusion pressure (CPPopt) can be calculated, i.e., the level of CPP where autoregulation functions best. The relation between cerebral blood flow (CBF) and CPPopt has not been examined. The objective was to assess to which extent CPPopt can be calculated in SAH patients and to investigate CPPopt in relation to CBF.

**Methods:**

Retrospective study of prospectively collected data. CBF was measured bedside with Xenon-enhanced CT (Xe-CT). The difference between actual CPP and CPPopt was calculated (CPP∆). Correlations between CPP∆ and CBF parameters were calculated with Spearman’s rank order correlation coefficient (rho). Separate calculations were done using all patients (day 0–14 after onset) as well as in two subgroups (day 0–3 and day 4–14).

**Results:**

Eighty-two patients with 145 Xe-CT scans were studied. Automated calculation of CPPopt was possible in adjunct to 60% of the Xe-CT scans. Actual CPP < CPPopt was associated with higher numbers of low-flow regions (CBF <10 ml/100 g/min) in both the early phase (day 0–3, *n* = 39, Spearman’s rho = −0.38, *p* = 0.02) and late acute phase of the disease (day 4–14, *n* = 35, Spearman’s rho = −0.39, *p* = 0.02). CPP level per se was not associated with CBF.

**Conclusions:**

Calculation of CPPopt is possible in a majority of patients with severe SAH. Actual CPP below CPPopt is associated with low CBF.

## Background

The ability to maintain stable cerebral blood flow (CBF) independently of variations in systemic blood pressure is referred to as cerebral pressure autoregulation (CPA). In aneurysmal subarachnoid hemorrhage (SAH), CPA may be disturbed to different extents [15]. Disturbed CPA in SAH is associated with delayed cerebral ischemia (DCI), vasospasm and unfavorable outcome [5, 7, 16]. CPA can be assessed with a number of methodologies, one of which is the pressure reactivity index (PRx) [9]. PRx is an autoregulatory index that relies on the correlation between spontaneous fluctuations in mean arterial blood pressure (MAP) and intracranial pressure (ICP). PRx has been studied extensively in traumatic brain injury (TBI) and to some extent also in SAH [2, 14].

Using PRx, optimal cerebral perfusion pressure (CPPopt) can be calculated. CPPopt is the level of cerebral perfusion pressure (CPP = MAP - ICP) within a given range where PRx is the lowest, i.e., where the status of autoregulation is optimal for the patient [26]. In TBI, patients with CPP close to their calculated optimum had better outcomes than those with large deviations, and treatment protocols focused on CPPopt have been proposed [1, 11, 29, 31]. Assessment of CPPopt is feasible also in patients with intracerebral hemorrhage and SAH, but the literature is limited, and there is a need for further studies to clarify the utility of CPPopt especially in SAH patients [3, 12, 22]. The aim of the present study was twofold: (1) to assess to which extent CPPopt can be calculated in SAH patients and (2) to investigate CPPopt in relation to cerebral blood flow (CBF) measured bedside with xenon-enhanced computed tomography (Xe-CT).

## Materials and methods

### Study design, patients and treatment protocol

The study was a retrospective analysis on prospectively collected data. SAH patients treated at the neurointensive care unit (NICU) at Uppsala University Hospital with valid CBF and PRx data were included. Data from day 0 to 14 after onset were accepted. As a consequence of the Xe-CT methodology we used, only patients with mechanical ventilation, i.e., severe SAH were included (see below).

Physiological variables (ICP, MAP and CPP) were recorded by a multimodality monitoring system and stored in a database for off-line calculations. A standardized treatment protocol was used, focusing on early detection and treatment of secondary insults [24]. Unconscious patients (GCS motor score ≤5) were intubated and mechanically ventilated, and received a ventriculostomy for ICP monitoring and cerebrospinal fluid (CSF) drainage. Propofol and morphine were used for sedation and analgesia, respectively. ICP and CPP targets were <20 mmHg and >60 mmHg. Other aims of the treatment protocol were normotension, normovolemia, body temperature <38 °C and normal electrolyte levels. Hydrocephalus and increased ICP were treated with CSF drainage against a pressure level of 15 mmHg. Aneurysms were treated early with endovascular coiling or surgical clipping.

### Xe-CT

In Xe-CT, inhaled stable xenon gas is used as a contrast agent [30]. Xenon is an inert, radiopaque gas that diffuses freely over the blood-brain barrier. A gas mixture with 28% xenon was delivered to the patient via the ventilator. After two unenhanced baseline scans, repeated scans of four 1-cm-thick slices with 1-cm gap were done during the wash-in phase of xenon. Quantitative CBF values were calculated from the dynamic changes in attenuation via a modified Kety-Schmidt equation. A system with a mobile CT scanner was used, which enables CBF measurement to be done bedside at the NICU. This was considered an advantage since severely ill patients may be impossible to transfer from the NICU for CBF measurements. The measured CBF values probably also better reflect the values during neurointensive care, rather than values measured at the radiology department, where ventilation and sedation parameters are likely to be different.

From the CBF raw data, three parameters were derived:

- Mean global cortical CBF
- Percentage of global cortical volume with CBF <20 ml/100 g/min (CBF% <20)
- Percentage of global cortical volume with CBF <10 ml/100 g/min (CBF% <10)

As a part of a standardized protocol, all intubated SAH patients were meant to undergo Xe-CT at day 0–1, day 4–7 and day 8–12 after onset of the disease. Scans could be added or omitted for clinical reasons. A common reason for not undergoing all three Xe-CT scans as planned was clinical improvement and extubation. Contraindications for Xe-CT include high oxygen demand and intractable intracranial hypertension.

### PRx

PRx was calculated as the moving correlation coefficient between PRx and MAP as described previously [4]. PRx is an index of autoregulation based on the relation between MAP and ICP and has been used extensively to study autoregulation in patients with traumatic brain injury, but also SAH [3, 5, 10]. PRx ranges between −1 and +1, negative or near-zero values signify intact autoregulation while positive values signify disturbed autoregulation.

### Optimal CPP

Optimal CPP was calculated automatically in a 4-h moving window that was updated every minute as described by Aries et al. [1]. CPP values ranging from 40 to 120 mmHg in the 4-h window were binned in intervals of 5 mmHg and values outside the range discarded. Corresponding mean PRx values were then plotted against CPP in the fixed 5 mmHg intervals. CPPopt was defined as the lowest PRx on a U-shaped curve. When a U-shaped curve could not be identified, the lowest PRx value on an ascending or descending curve was accepted, given that the curve follows a convex shape, and that the ascending curve was at the lower end of the CPP interval, or the ascending curve at the higher end of the CPP interval. If the criteria of the automatic algorithm were not fulfilled, CPPopt was not calculated for that minute.

The difference between calculated CPPopt and actual CPP (CPPΔ) was calculated as the difference between CPPopt and mean CPP during 30 min before Xe-CT. Positive CPPΔ means that the actual CPP was higher than calculated optimal CPP, and vice versa.

### Statistical analyses and patient subgroups

Correlations between CPPΔ and CBF parameters were assessed with Spearman’s rank order correlation coefficient (rho). Non-parametric testing was used because of non-normality in the CBF parameters (assessed by visual inspection of the raw data) and because linear relationships could not be assumed. Three calculations were done: one using all data (day 0–14) and two subgroups using data only from day 0–3 to day 4–14, respectively. The rationale behind the subgrouping is the occurrence of vasospasm, which is seen in about 2/3 of SAH patients with the highest prevalence on day 4–14 [20, 21]. Vasospasm may affect CPA [7] and potentially also influence PRx and CPPopt calculations [18]. The two subgroup time windows correspond approximately to before and during the expected peak prevalence of vasospasm.

Only one Xe-CT per patient in each time period was used in the calculations. If a patient was scanned multiple times in the same time window, only data from one scan was used, and the first scan with valid CPPopt data was chosen primarily. Since CPP will be a determinant in CPPΔ, correlations between CPP (mean CPP 30 min before Xe-CT) and CBF parameters were also calculated to assess whether any correlations between CPPΔ and CBF parameters were attributed only to level of CPP.

## Results

One-hundred forty-five Xe-CT scans were performed on 82 SAH patients with PRx data treated at the neurointensive care unit, Uppsala University Hospital, between October 2012 and January 2016 (median number of Xe-CT scans per patient = 2, range 1–4). Sixty-six percent were female, and mean age was 59.7 years (range 28–84 years). Median CT Fisher score was 4 (range 2–4) and median Hunt & Hess grade was 3 (range 1–5). Sixty-seven patients (82%) were treated with endovascular coiling and 15 patients (18%) with surgical clipping. Some patients with low Hunt & Hess grade at admission later deteriorated and were intubated and could be included in the study. Median and interquartile ranges of the CBF parameters are presented in Table 1.

**Table 1:** Median (interquartile range) of mean global CBF, CBF% <10 and CBF% <20 in the different time windows used in this study

Time window	Mean global CBF ml/100 mg/min Median (interquartile range)	CBF% <10 No unit Median (interquartile range)	CBF% <20 No unit Median (interquartile range)
Day 0–14 (*n* = 64)	31.5 (25.0–39.0)	1.6 (0.0–6.7)	16.1 (4.3–34.8)
Day 0–3 (*n* = 39)	32.9 (26.6–40.1)	0.0 (0.0–6.7)	12.9 (3.3–30.4)
Day 4–14 (*n* = 35)	29.5 (24.8–38.3)	1.8 (0.0–5.0)	18.8 (3.3–35.0)

CPPopt could be calculated in adjunct to 87 of the 145 Xe-CT scans (60%) in 64 of the 82 patients (78%). Of the 64 patients, 39 were examined during day 0–3 and 35 patients during day 4–14 (10 patients examined in both periods). In the patients where CPPopt could be calculated, the median and interquartile range of CPPopt, actual CPP and CPPΔ in the different time windows are presented in Table 2. Negative CPPΔ indicates actual CPP < CPPopt, and vice versa.

**Table 2:** Median (interquartile range) of CPPopt, actual CPP (30 min mean before Xe-CT) and in the different time windows used in the study

Time window	CPPopt, median (interquartile range)	Actual CPP, median (interquartile range)	CPPΔ, median (interquartile range)
Day 0–14 (*n* = 64)	80.0 (72.0–88.0)	76.9 (69.7–83.0)	−3.9 (−11.2–7.9)
Day 0–3 (*n* = 39)	77.0 (71.0–86.0)	74.7 (69.1–83.0)	−3.9 (−6.6–8.0)
Day 4–14 (*n* = 35)	81.0 (73.0–93.0)	79.8 (71.5–85.1)	−3.0 (−12.4–6.3)

Negative CPPΔ indicates actual CPP < calculated CPPopt and vice versa

### Association between CPPΔ and CBF parameters

**Fig. 1:**
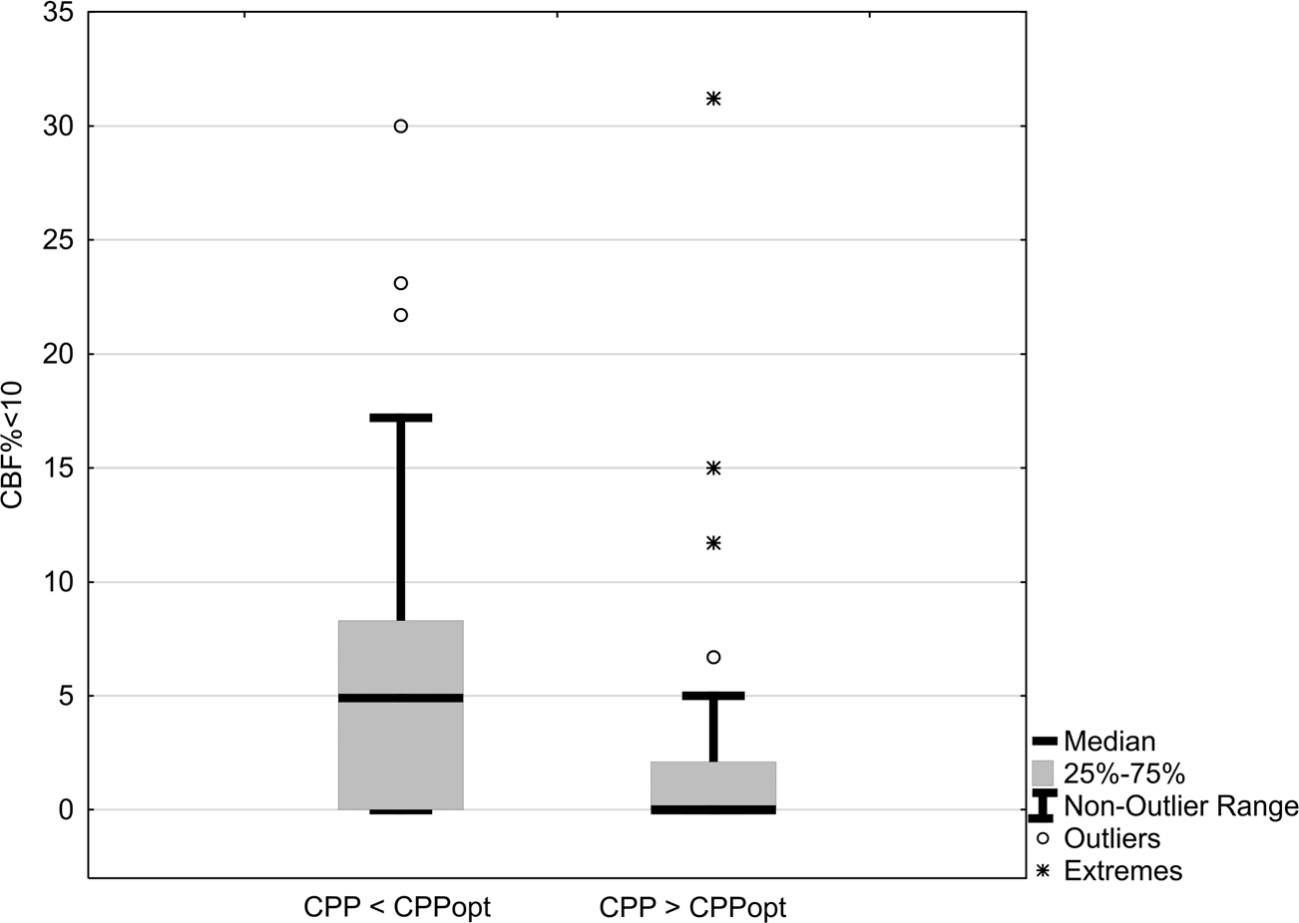
Patients with actual CPP < calculated optimal CPP had higher numbers of regions with CBF <10 ml/100 g/min. *p* = 0.008, Mann-Whitney *U*-test. Time window = day 0–14, *n* = 64

Day 0–14:

CPPΔ was moderately negatively correlated with CBF% <10, CBF% <20 and mean global CBF with borderline significance. Mean CPP was not correlated with any of the CBF parameters (Table 3). Patients with actual CPP below CPPopt had significantly higher amounts of CBF% <10 (median 5% vs. 0%, *p* = 0.008, Mann-Whitney *U*-test, Fig 1).

**Table 3:** Correlations between CPPΔ/mean CPP (30 min mean before Xe-CT) and CBF parameters

	CPPΔ		Actual CPP	
	Rho	p	Rho	p
Mean global CBF	0.24	0.05	−0.08	0.51
CBF% <10	−0.39	0.002	0.01	0.94
CBF% <20	−0.27	0.03	0.11	0.37

Time window = day 0–14, *n* = 64. Rho, *p* = Spearman’s rank order correlation

Day 0–3:

CPPΔ was moderately negatively correlated with CBF% <10. Mean CPP was not correlated with any of the CBF parameters (Table 4).

**Table 4:** Correlations between CPPΔ/mean CPP (30 min mean before Xe-CT) and CBF parameters

	CPPΔ		Actual CPP	
	Rho	p	Rho	p
Mean global CBF	0.15	0.37	−0.22	0.18
CBF% <10	−0.38	0.02	0.02	0.88
CBF% <20	−0.19	0.25	0.21	0.20

Time window = day 0–3, *n* = 39. Rho, *p* = Spearman’s rank order correlation

Day 4–14:

CPPΔ was moderately negatively correlated with CBF% <10. Mean CPP was not correlated with any of the CBF parameters (Table 5).

**Table 5:** Correlations between CPPΔ /mean CPP (30 min mean before Xe-CT) and CBF parameters

	CPPΔ		Actual CPP	
	Rho	p	Rho	p
Mean global CBF	0.29	0.09	0.23	0.18
CBF% <10	−0.39	0.02	−0.12	0.51
CBF% <20	−0.30	0.08	−0.03	0.87

Time window = day 4–14, *n* = 35. Rho, *p* = Spearman’s rank order correlation

## Discussion

### Main findings

This is, to our knowledge, the first study to investigate the relation between CPPopt and CBF in SAH patients. CPPopt could be calculated in adjunct to at least one Xe-CT scan in 78% of the patients and in 60% of all Xe-CT scans performed, using data from a 4-h time window. The fact that CPPopt could not be calculated in all patients is of course a limitation, but the numbers are reasonably high to be of use clinically. ΔCPP was negatively correlated with CBF% <10 and CBF% <20, meaning that patients with actual CPP < calculated CPPopt had higher numbers of low-flow regions (Table 3, Fig 1). The effect was not attributed to the CPP level per se, as CPP itself was not correlated with any of the CBF parameters in any of the time windows (Tables 2 and 3). This suggests that CPP in SAH patients should be individualized according to the status of autoregulation and that low CPP in relation to the calculated optimum should be avoided. The findings were consistent in both the early (day 0–3) and late (day 4–14) time windows, but were more clear when data from all monitoring times were used, possibly because of the larger sample size. In the two time windows with smaller sample size, actual CPP above CPPopt was associated with a lower number of regions with CBF <10 ml/100 g/min. This is clinically important since CBF 10 ml/100 g/min is very close to ischemic levels [19, 30].

The correlations found were only weak to moderate, and CBF is of course determined by many factors other than the difference between CPP and CPPopt. CPPopt however seems to be important to consider in the clinical setting since it provides an available measure that may be possible to use to individualize treatment according to the status of autoregulation.

It appears from the results of this study that keeping the actual CPP at the level of calculated CPPopt or even higher reduces the risk of regional hypoperfusion at critical CBF levels (<10 ml/100 g/min), while actual CPP levels below the calculated CPPopt increase the risk. The lack of association between CPP values per se and CBF may of course be a statistical artifact (type II error), but suggests that CPP need not be high, as long as it is high enough for the individual patients. It should also be noted that the dispersion of CPP values was not great (Table 1), and if low CPP values were more common, there would probably have been a correlation between CPP per se and CBF.

Since the present study was not an outcome study, the effect of the relation between CPP and CPPopt on clinical outcome is not known. Hypoperfusion after SAH has however been associated with unfavorable outcome, and optimization of CBF is probably of great importance in the clinical management [25].

### Methodological issues

When calculating CPPopt, a few methodological aspects are important to consider. One is craniospinal compliance. In situations with high craniospinal compliance (large capacity to compensate for added intracranial volume), the relation between changes in cerebral blood volume (vasodilatation and vasoconstriction) and ICP may be less pronounced. Changes in MAP will then not be reflected in ICP changes regardless of the status of pressure autoregulation, and PRx will be less reliable. This may be the case after decompressive craniectomy or when CSF is drained via an open ventricular catheter drainage system. In the ventricular drainage system used at our institution, however, a small rubber valve at the outflow tube keeps the CSF from flowing freely, and the oscillations of the ICP curve are still visible even with an open drain. There is evidence that PRx calculations are valid with open ventricular drains as long as the ICP curve has a normal configuration [1]. Furthermore, differences found in PRx calculated with open/closed ventricular drains are probably small and clinically insignificant [17]. In this study, we therefore opted to use PRx data also from time periods with open ventricular drains.

Another aspect is the time window used to calculate CPPopt. CPPopt calculated from long time periods (i.e., data from all monitoring time) produces robust associations with outcome [26]. The status of autoregulation, and therefore optimal CPP, may however be different at different time points [27]. This approach is therefore not applicable if CPPopt is to be used for clinical decision-making. In this study we used a 4-h moving time window for CPPopt calculations. This approach has proved feasible in TBI and may be valuable in the clinical setting for continuous monitoring and customization of treatment [1].

A third methodological aspect is the algorithm used to calculate CPPopt. Different methods have been described [1, 26, 31]. In the original report by Steiner et al., a U-shaped curve was required, and CPPopt was taken as the lowest point on that curve [26]. This method allowed for estimation of CPPopt in 60% of patients, which is in accordance with the results of this study. Other approaches have allowed different curve shapes as well or simply taken CPPopt as the lowest PRx in a given CPP interval [1, 31]. In this study, CPPopt was taken as either the lowest PRx on a U-shaped curve or the lowest PRx on an ascending or descending convex curve. In case of an ascending or descending convex curve, it was assumed that the range of CPP was too small to form a U-shaped optimization curve, and a U-shaped curve would be found with a wider CPP range.

Another consideration is how vasospasm may affect PRx. Vascular constriction at different levels of the vascular tree may affect autoregulation in different ways. The calculation of PRx may also be affected in an unpredictable way, and the PRx values may be invalid with both false high and low values making interpretation difficult [18]. Calcium antagonists (Nimodipine) are routinely used in SAH and improve outcome [13]. However, its impact on autoregulation is not clear. Calcium antagonists have a direct effect on smooth muscle cells and in one study on healthy subjects have been shown to affect CBF and flow velocity in intracerebral arteries [28]. Another study on SAH patients found that Nimodipine affected one autoregulatory index (ORx) but not PRx [8]. A limitation of the present study is that vasospasm was not assessed routinely with transcranial Doppler or angiography. The effect of vasospasm on the measurement of CPPopt could therefore not be evaluated. However, it is unlikely that vasospasm occurred during the early period day 0–3 and the results were consistent for all periods.

Retrospective studies, such as the present, are susceptible to bias. For example, the inclusion of subjects may be different if subjects are recruited continuously by predetermined criteria. Confounding is another common problem in retrospective studies, i.e., the effects are caused by unknown factors that are not measured. Since the data in this study were collected systematically in a prospective fashion and not known to the investigators at the time of study design, some of the inherent limitations of a retrospective study are overcome.

Finally, studies with a small sample size, such as the present study, are common in clinical research and have some caveats regarding significance testing. A reduced ability of detecting a true effect is intuitive, but there is also an increased risk of significant findings being false positives [6, 23]. Increasing the statistical power by increasing the sample size decreases these risks. In clinical research, however, this is not always possible. The present study is to be considered hypothesis generating rather than hypothesis testing. The statistical methods were chosen to be as robust as possible, and we do not make any claims about effect sizes. The results must still be interpreted with some caution.

## Conclusions

Calculation of CPPopt was possible in clearly more than half of the SAH patients included in the study (60% of all examinations and 78% of the examined patients) using data from both open/closed ventricular drains.

Actual CPP below CPPopt is associated with low CBF.

Taken together, the present study demonstrates the physiological CBF properties of CPPopt and that CPPopt may be a valuable tool to monitor SAH patients and individualize CPP treatment to avoid regional hypoperfusion. Further studies are warranted to evaluate the utility of CPPopt in the clinical management of SAH patients.
